# Characterization of Alternative Splicing (AS) Events during Chicken (*Gallus gallus*) Male Germ-Line Stem Cell Differentiation with Single-Cell RNA-seq

**DOI:** 10.3390/ani11051469

**Published:** 2021-05-20

**Authors:** Changhua Sun, Kai Jin, Qisheng Zuo, Hongyan Sun, Jiuzhou Song, Yani Zhang, Guohong Chen, Bichun Li

**Affiliations:** 1 Key Laboratory of Animal Breeding Reproduction and Molecular Design for Jiangsu Province, College of Animal Science and Technology, Yangzhou University, Yangzhou 225009, China; echo7260@163.com (C.S.); jinkai0621@163.com (K.J.); 006664@yzu.edu.cn (Q.Z.); hongyans2392@163.com (H.S.); ynzhang@yzu.edu.cn (Y.Z.); ghchen@yzu.edu.cn (G.C.); 2 Department of Food Technology, College of Biochemical Engineering, Yangzhou Polytechnic College, Yangzhou 225009, China; 3 Joint International Research Laboratory of Agriculture and Agri-Product Safety of the Ministry of Education of China, Yangzhou University, Yangzhou 225009, China; 4 Animal & Avian Sciences, University of Maryland, College Park, MD 20741, USA; songj88@umd.edu

**Keywords:** alternative splicing (AS) events, chicken (*Gallus gallus*), male germ cell differentiation, single-cell RNA-seq

## Abstract

**Simple Summary:**

Studies have shown that alternative splicing (AS) has been utilized in a wide variety of life processes. However, there are very few studies on AS during germ cell development. In this study, we preliminarily investigated the variation of variable shear events during the formation of chicken germ cells through the RNA-seq data analysis of embryonic stem cells (ESCs), gonad PGCs (gPGCs), and spermatogonia stem cells (SSCs), and the critical AS mode for several crucial stage-specific genes, which were identified during germ cell development. The results of this study lay a theoretical foundation for further analysis of the regulation mechanism of key genes involved in germ cell formation.

**Abstract:**

Alternative splicing (AS) is a ubiquitous, co-transcriptional, and post-transcriptional regulation mechanism during certain developmental processes, such as germ cell differentiation. A thorough understanding of germ cell differentiation will help us to open new avenues for avian reproduction, stem cell biology, and advances in medicines for human consumption. Here, based on single-cell RNA-seq, we characterized genome-wide AS events in manifold chicken male germ cells: embryonic stem cells (ESCs), gonad primordial germ cells (gPGCs), and spermatogonia stem cells (SSCs). A total of 38,494 AS events from 15,338 genes were detected in ESCs, with a total of 48,955 events from 14,783 genes and 49,900 events from 15,089 genes observed in gPGCs and SSCs, respectively. Moreover, this distribution of AS events suggests the diverse splicing feature of ESCs, gPGCs, and SSCs. Finally, several crucial stage-specific genes, such as NANOG, POU5F3, LIN28B, BMP4, STRA8, and LHX9, were identified in AS events that were transmitted in ESCs, gPGCs, and SSCs. The gene expression results of the RNA-seq data were validated by qRT-PCR. In summary, we provided a comprehensive atlas of the genome-wide scale of the AS event landscape in male chicken germ-line cells and presented its distribution for the first time. This research may someday improve treatment options for men suffering from male infertility.

## 1. Introduction

Alternative splicing (AS) is an important, co-transcriptional, and post-transcriptional regulatory mechanism that results in multiple mRNA and protein isoforms from a single gene. Protein isoforms show different or mutually antagonistically functional and structural characteristics, which play critical roles in cellular processes, such as cell signaling and differentiation [1,2,3]. Recent high-throughput analyses have shown their ubiquitousness in nematode worm genes (*Caenorhabditis elegans*, 71%), human genes (*Homo sapiens*, 95%), mouse genes (*Mus musculus*, 57%), cow genes (*Bos taurus*, 21%), etc. [4,5,6]. The classical AS event types include the alternative 5′ splice site (A5′), alternative 3′ splice site (A3′), exon-skipping (ES), intron retention (IR), and alternative exon (AE), which were involved in the mRNA transcription from one gene via the spliced site of pre-mRNA [7]. Therefore, AS is the fundamental cause of significantly fewer genes than protein species in eukaryotes. Generally, AS events are involved in many physiological processes, as well as cell differentiation, apoptosis, and development. Abnormal AS events will cause deviant processes by changing gene expression products [8,9,10,11]. Hence, studying AS in biological processes is vitally important and essential.

Germ cells are exclusive cells which are capable of bringing genetic information to the next generation and sustaining life. Their regulation processes are subtle and intricate during cell differentiation [12,13,14]. It is noted that the formation, migration, and differentiation of germ cells are current hotspots in animal genetics, breeding, and reproduction research [15,16,17]. Moreover, germ cells can be isolated, cultured, and substituted for embryonic stem cells (ESCs) in the treatment of diseases (such as infertility and organ regeneration) without ethical and immune rejection complications [18,19]. Especially in chickens (*Gallus gallus*), the unique migration pattern of primordial germ cells (PGCs) and advantages of embryonic development *in ovum* make germ cell isolation easier, and abundant germ cells could be applied to advanced stem cell research and transgenic animal production [20,21]. In addition, chickens are a classic avian experimental model, and they have been used extensively in developmental animal biological research [22,23]. For these reasons, understanding of chicken germ cell biology has not only accelerated the practical application of avian reproduction biology and fundamental stem cell research, but also assisted human medicine and medical applications (including various birth defects, germ cell tumor, and drug target screening). The utilization of these methods allows researchers and scientists to avoid the ethical constraints of other methods [24]. Hence, the study of chicken germ cell differentiation is an extremely urgent need.

Recent evidence suggests that AS events are an essential and vital factor in developmental biology and have been shown to play important roles in testis proteome diversification and male germ cell differentiation via numerous mechanisms [25,26,27]. However, there has been a scarcity of reports regarding AS events studied in chicken germ cells, and the distribution and features of these reports are still unclear. Now, single RNA-seq and relevant bioinformatic analysis methods are powerful and accurate tools to characterize AS events in an entire genome [28,29]. Therefore, we screened relevant AS events related to the differentiation of chicken ESCs, gPGCs, and spermatogonia stem cells (SSCs) through single-cell RNA-seq. The primary aim of our study was to determine the distribution of AS events that participate in the development and differentiation of male germ cells. We went further by analyzing AS event genes to construct an interaction network and identify key pathways in bioinformatics to sharpen our insight into reinterpreting specific pathways. Finally, we investigated AS events and expression patterns of key genes in ESCs, gPGCs, and SSCs. This study not only provides a comprehensive view of AS events in chickens, but also highlights novel insights into potential roles of AS events in the study of male germ cell differentiation.

## 2. Materials and Methods

### 2.1. Materials

Chicken eggs were collected from Rugao Yellow chickens (from the Poultry Institute, Chinese Academy of Agricultural Sciences, Yangzhou, China). All eggs were incubated at 37 °C and 75% relative humidity for 4.5 days or 18.5 days.

### 2.2. Cells and RNA-seq

The method of chicken ESC, gPGC, and SSC isolation referred to the previous report [30,31]. Briefly, ESCs was separated from newly laid fertilized eggs (blastoderm, E0, HH stage X) and digested with trypsin-EDTA (0.25%) for 3 min. gPGCs were isolated from eggs which had been incubated for 4.5 days (gonads, E4.5, HH stage 28–30). Genital ridges from embryos were collected and cut up, followed by digestion with trypsin-EDTA (0.25%). SSCs were isolated from eggs which had been incubated for 18.5 days (testis, E18.5, HH stage 44)). Testes from male embryos were collected and cut up, followed by the digestion with Collagenase I and trypsin-EDTA (0.25%). Isolated cells were filtered and prepared for further RNA extraction.

Cells were collected and then treated with a SMART-Seq v4 ultra low input RNA kit for sequencing (Takara, Dalian, China, 634850). The RNA from each sample was used for cDNA synthesis and sequencing. The RNA library pools for three kinds of cells were established following the protocol of *Illumina Hiseq2000 PE150*, and the experiments were performed at the Novogene Company (Beijing, China).

### 2.3. RNA-Seq Reads Mapping

Filtering and quality control checks of raw reads from RNA-seq were performed utilizing the Fast QC program (http://www.bioinformatics.babraham.ac.uk/projects/fastqc/, accessed on 1 August 2019). The clean reads were mapped to the chicken reference genome (*Gallus gallus*, galGal4.0) using Tophat (version 2.1.1) [32]. The mapped reads were assembled into contigs with Cufflinks (version 2.2.1). The expression value for each gene was calculated and normalized to FPKM (fragments per kilobase of exon per million fragments mapped) values using Cuffnorm (version 2.2.1) in the Cufflinks package [33].

### 2.4. Identification, Classification, and Quantification of AS Events

After utilizing *Cufflinks*, the identification and quantification of AS events was accomplished using *ASprofile* [34]. Initially, the extract AS program compares all pairs of transcripts within a gene to determine exon–intron structural differences that indicate an AS event. The following types of events have been implemented: exon skipping (SKIP), cassette exons (MSKIP), alternative transcript start and termination (TSS, TTS), retention of single or multiple introns (IR, MIR), and alternative exons (AEs). Additionally, variations on these classes have allowed for some wiggle room at the boundaries of the surrounding features, and they have been reported and prefixed with an X. Therefore, we classified these AS events into 5 types: alternative 5′ splice Site (A5′, including TSS and XTSS), alternative 3′ splice Site (A3′, including TTS and XTTS), exon skip (ES, including SKIP, MSKIP, XSKIP, and XMSKIP), retained intron (RI, including IR, XMIR, XIR, and XMIR), and alternative exon (AE, including AE and XAE). Finally, the *extract-as-fpkm program* calculates the fragments per kilobase million (FPKM) of each event from those of transcripts harboring an event in each sample. The results can be used to observe trends in dynamics of AS profiles.

### 2.5. Intersections and Interaction Network Analysis of the AS Event Gene

The intersections (Upset plot and Veen plot) between different types of AS events were also investigated and illustrated by employing the UpSetR package (version 1.4.0, https://www.r-project.org/web/packages/UpSetR, accessed on 22 May 2019) of R software (version 3.6.2, https://www.r-project.org/, accessed on 12 December 2019). After selection by Veen plot, the genes of AS events were submitted to String (version 11.0, https://string-db.org/, accessed on 19 January 2019) for the protein–protein interaction (PPI) analysis. Cystoscope (version 3.7.2, https://cytoscape.org/, accessed on 5 September 2019) was then applied to illustrate and identify neighborhoods where proteins were densely connected in the PPI network.

### 2.6. GO Enrichment and KEGG Pathway Analysis of AS Events Gene

Gene ontology (GO) enrichment analysis was performed using *DAVID* (v6.8, https://david.ncifcrf.gov/, accessed on 1 September 2020) [35,36], and GO terms were considered as statistically significant with *p*-values < 0.05. GO terms have been classified by the GO database (ftp://ftp.ncbi.nih.gov/gene/DATA/gene2-go.gz, accessed on 10 May 2017), which is an international standard gene functional classification system. In addition, BinGO, which illustrates relationships among GO terms, was explained using Cytoscape 3.7.2 version [37]. The Kyoto Encyclopedia of Genes and Genomes (KEGG) helps researchers to better understand the biological functions of genes based on large-scale molecular datasets (http://www.genome.jp/kegg/, accessed on 10 May 2017). In this study, the enrichment of KEGG was analyzed by KOBAS 3.0 (http://kobas.cbi.pku.edu.cn/kobas3/annotate/, accessed on 20 September 2018) and a *p*-value < 0.05 was set as the cutoff for significantly enriched *KEGG* terms. Illustrations were performed through the ggplot2 package in R [38].

### 2.7. RNA Preparation and RT-qPCR

Isolated cells were homogenized using TRIzol, and the total RNA was isolated according to the manufacturer’s instructions (QIAGEN, Beijing, China, DP424). qRT-PCR was performed using the FastKing One-Step RT-PCR Kit with SYBR green (QIAGEN, Beijing, China, KR123), and the mRNA levels were determined by a CFX-Connect Real-time PCR detection system (BIO-RAD, Hercules, CA, USA, 7500fast). Quantification results were normalized to the relative housing keeping gene (GAPDH) using the 2-^ΔΔCt^ method. The sequences of qRT-PCR primers are listed in Table 1.

## 3. Results

### 3.1. The AS Events during Male Chicken Germ Cell Differentiation

**Figure 1:**
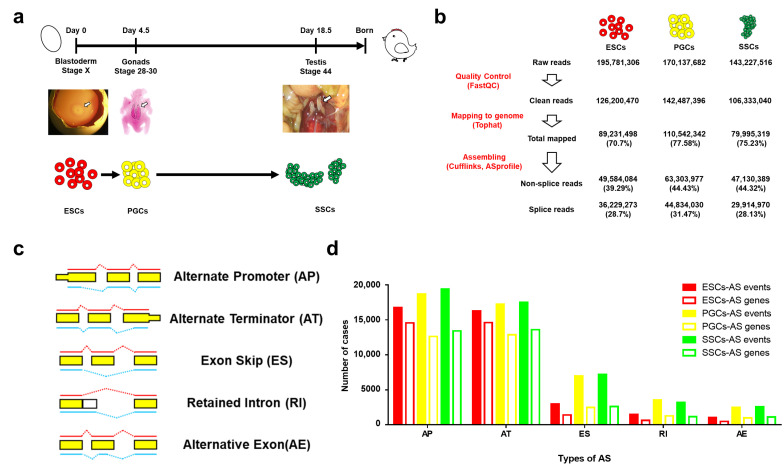
AS events during chicken male germ cell differentiation. (**a**) ESCs were isolated from the blastoderm of fertile eggs at E0 (HH stage X). gPGCs were isolated from chicken gonads at E4.5 (HH stage 28–30), and SSCs were isolated from chicken testis at E18.5 (HH stage 44). (**b**) The overview of RNA-seq data analysis pipeline. (**c**) Representative model for five types of AS events, including alternative 5′ splice site (A5′), alternative 3′ splice site (A3′), exon skip (ES), retained intron (RI), and alternative exon (AE). (**d**) Numbers of AS events and involved genes during male chicken germ cell differentiation.

To study AS events in male chicken germ cell differentiation, we performed genome-wide RNA-seq for ESCs, gPGCs, and SSCs isolated from chicken embryonic blastoderms (E0, HH stage X), gonads (E4.5, HH stage 28–30), and testes (E18.5, HH stage 44), respectively (Figure 1a). Our results showed that the sequencing of the three cells transcriptome provided 195.7 million reads of ESCs, 170.1 million reads of gPGCs, and 143.2 million reads of SSCs. After quality control and mapping to the entire annotated chicken genome (*galGal* 4.0), 28.7%, 31.47% and 28.13% mapped splice reads were observed in ESCs, gPGCs, and SSCs, respectively (see Figure 1b). To refine and explore AS event changes during germ cell differentiation, we divided AS events into alternative 5′ splice site (A5′), alternative 3′ splice site (A3′), exon skip (ES), retained intron (RI), and alternative exon (AE) (see Figure 1c). In ESCs, a total of 38,494 AS events from 15,338 genes were detected, indicating that each gene might have had 2–3 AS events on average. However, 48,955 AS events from 14,783 genes and 49,900 AS events from 15,089 genes were found in gPGCs and SSCs, respectively. This indicated an average of 3–4 AS events from each gene, which was a notably higher frequency than that in ESCs (see Table 2). In detail, most AS events detected during germ cell differentiation were A5′ and A3′ followed by ES, RI, and AE, which further implies that gPGCs and SSCs had a higher frequency of AS events than ESCs across all five kinds of AS events, especially ES, RI, and AE (see Figure 1d). Moreover, ES, RI, and AE were much less prevalent than A5′/A3′ in three germ cell types, which suggests that A5′/A3′ are major AS events during chicken male germ cell differentiation.

### 3.2. AS Event Distribution Analysis during Male Chicken Germ Cell Differentiation

**Figure 2:**
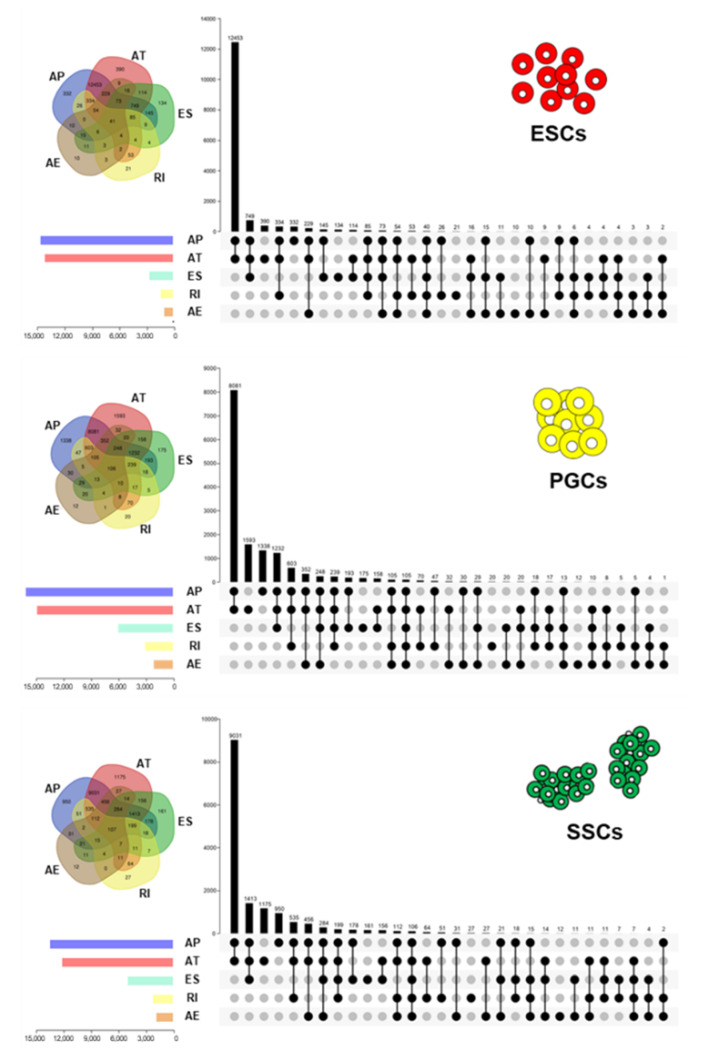
Distribution analysis of AS events during male chicken germ cell differentiation. The Veen and Upset plots show the distribution of AS events in chicken ESCs, gPGCs, and SSCs. The bar chart above represents the number of genes contained in each type of group. The bar chart at the bottom-left represents the number of events included in each type of AS event. The dotted line at the bottom-right shows the types of events contained in the group.

To investigate the splicing feature of each germ cell, we generalized the common gene distribution of AS events during male germ cell differentiation. The results showed that the A5′/A3′ gene group contained 12,453, 8081, and 9031 genes in ESCs, gPGCs, and SSCs, respectively. This was the intersection group with the largest number of genes involved in two types of events. However, the A5′/A3′/ES and AT groups contained 749 and 390 genes which followed the A5′/A3′ group in ESCs. In gPGCs, the following groups were the A5′/A3′ groups, which contained 1593 and 1338 genes, respectively. Additionally, SSCs, accounting for 1413 and 1175 genes, were contained in the A5′/A3′/ES group, and the A3′ group followed the A5′/A3′ group. In each type of male germ cell, different splicing features were present; more details can be found in Figure 2 and Tables S1–S3. Overall, the male germ cell exhibited different distribution characteristics of AS events, suggesting that AS is involved in the male germ cell differentiation.

### 3.3. A Cell-Type-Specific Spliced Gene in ESCs, GPGCs, and SSCs

**Figure 3:**
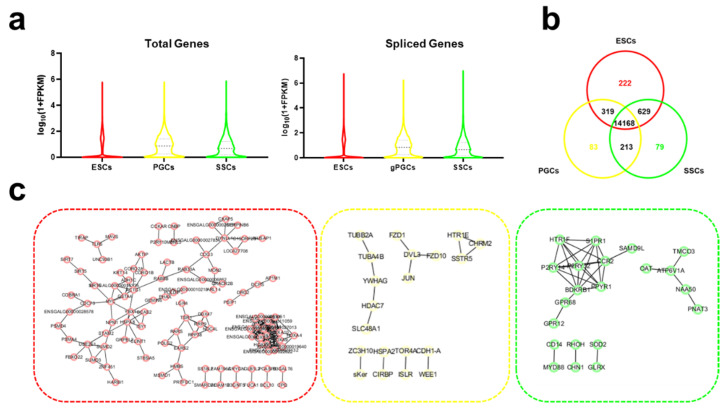
A cell-type-specific spliced gene in ESCs, gPGCs, and SSCs. (**a**) The expression patterns of total genes and spliced genes during male chicken germ cell differentiation. (**b**) Differentially spliced genes among three cell types. (**c**) The gene network of cell-specific spliced gene groups. The red dotted line is the ESC-specific spliced gene–gene interaction network; the yellow dotted line is the gPGC-specific spliced gene–gene interaction network; and the green dotted line is the gPGC-specific spliced gene–gene interaction network.

To find out the potential influence of AS in male germ cell gene expressions, we compared the total gene and spliced gene expression patterns in ESCs, gPGCs, and SSCs. They shared a similar expression pattern (see Figure 3a; Tables S4 and S5). In addition to the expression pattern comparison at the gene level, we studied differentially spliced genes during the male germ cell differentiation and compared AS genes of three germ cells. Their overlap had 14,168 genes, and 222, 83, and 79 of these genes were singlehandedly present in ESCs, gPGCs, and SSCs, respectively (see Figure 3b). To determine whether the genes were affected by male germ cell differentiation, we analyzed these cell-specific gene groups. The network results revealed that ESCs might contain four interaction networks, which include hub genes at MYC, HOXA, RARS, etc. However, the GPGCs and SSCs may exhibit fewer networks and hub genes (see Figure 3c). Taken together, these results imply that differentially spliced genes appear in male germ cell differentiations.

### 3.4. Function Enrichment Results of Spliced Genes during Male Germ Cell Differentiation

**Figure 4:**
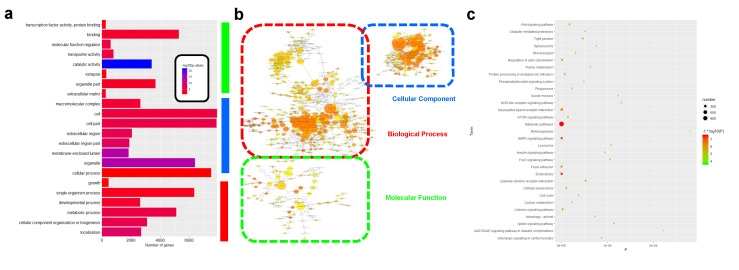
Functional enrichment analysis results of spliced genes during male germ cell differentiation. (**a**) The significantly enriched (*p* < 0.05) first-class GO terms cluster of AS genes that were alternatively spliced in all cell types. The red line represents the biological process terms; the blue line represents cellular component terms; and the green line represents molecular function terms. (**b**) The enriched GO terms network. Each term is represented by a circle node, where its size is proportional to the number of AS genes, and its color represents its cluster identity. (**c**) The top 30 enriched KEGG pathway of AS genes that were alternatively spliced in all cell types.

To verify the function of these spliced genes, we performed GO and KEGG pathway enrichment analyses universally for the process of spliced genes during male germ cell differentiation. In total, 22 first-class GO terms were significantly identified: 7 GO terms were enriched in biological processes, including cellular process (GO:0009987), developmental process (GO:0032502), and metabolic process (GO:0008152). Additionally, we found 10 and 5 GO terms in the cellular component and molecular function parts, respectively (see Figure 4a, Table S6). Moreover, GO terms such as cellular process (GO:0009987), developmental process (GO:0032502), and metabolic process (GO:0008152) stand out, which play important roles in the biological process (Figure 4b). A total of 166 KEGG pathways were significantly enriched by the overall spliced genes (Table S7). The top 30 pathways are shown in Figure 4c, containing metabolic pathways (gga01100), endocytosis (gga04144), MAPK signaling pathway (gga04010), calcium signaling pathway (gga04218), Wnt signaling pathway (gga04310), and spliceosome (gga03040) (Figure 4c). These results indicate that spliced genes widely exist in the cell differentiation process.

### 3.5. Validation of AS Events and Crucial Gene Expression

**Figure 5:**
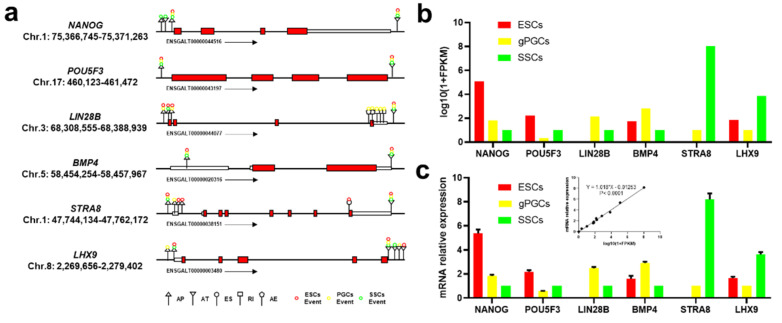
Validation of AS events and crucial gene expression. (**a**) The gene transcript structure and AS events of crucial genes in male chicken germ cell differentiation. The red square is exon, the different color cycles are AS events, and the purple arrow represents the qRT-PCR primers at the constitutive exon loci. (**b**) The gene expression of RNA-seq data. (**c**) The gene expression of qRT-PCR results and correlation analysis.

Even previous results showed that spliced genes have similar expression patterns to total genes, but details of the relationship between AS events and spliced genes are still unknown. We displayed AS events from six genes which play a crucial role in male chicken germ cell differentiation. NANOG and POU5F3, highly expressed in ESCs, exhibited A5′ and A3′ events [39,40]. The LIN28B had more types of AS events in gPGCs, and both of the BMP4 had specific expression in gPGCs [41,42] (see Figure 5a). STRA8 and LHX9 exhibited high expressions in SSCs and showed different patterns of AS events during male germ cell differentiation [43,44] (see Figure 5b). Transcript levels of these genes in ESCs, gPGCs, and SSCs were measured by qRT-PCR. The consistent results from both qRT-PCR and RNA-Seq suggested the reliability of RNA-Seq data and its accuracy in quantifying gene expressions in male germ cells (see Figure 5c).

## 4. Discussion

The phenomenon of AS was discovered in the twentieth century, but it has not been systemically analyzed [45]. Advances in high-throughput sequencing provide an efficient technology to acquire AS events at the genome-wide level [46]. In this study, we reported and characterized genome-wide AS events during male chicken germ cell differentiation through a systemic comparison of single-cell RNA-seq. Results showed that AS events, especially A5′/A3′ events, are comprehensively involved in the process of male germ cell differentiation, while showing different patterns in ESCs, gPGCs, and SSCs. Functional enrichments in GO and KEGG pathways are closely related to cell differentiation. Moreover, some crucial gene expression patterns are differential AS events. Collectively, all these results provide a glimpse of dynamic involvements and systematical profiling of AS events in chicken ESCs, gPGCs, and SSCs, which might arouse the research use of numerous, potential biomarkers and AS events in the study of male germ cell differentiation.

A diverse splicing pattern in one gene leads to a variety of isoforms and expression patterns, which makes AS and its regulation mechanism more complex in the germ cellular process [47]. In this study, a total of 38,494 AS events out of 15,338 genes in ESCs, 48,955 AS events out of 14,783 genes in gPGCs, and 49,900 out of 15,089 genes in SSCs were detected. This is significant, in that AS is a commonly used process during male chicken germ cell differentiation. In addition, all cells generated the largest number of A5′ and A3′, while the smallest number of AEs was generated. It is possible that the A5′/A3′ is major factor in determining the transcript splicing, thus each alternative transcript is somewhat defined by the A5′/A3′. After comparing the difference of AS preference through ESCs, gPGCs, and SSCs, five types of AS events were increased, and ES, RI, and AE were all significantly enhanced in gPGCs and SSCs. The difference in AS has also been described in mammalian spermatogenesis, giving support to the agreement that changes in AS may contribute to male germ cell differentiation [48,49]. Moreover, the distribution of AS events demonstrated differential AS event patterns in ESCs, gPGCs, and SSCs. It is obvious that the splicing features of AS events may lead to direct differentiation.

To further understand the gene function affected by AS through influencing male chicken germ cell differentiation, we performed PPI, GO, and KEGG analyses of AS genes, and found that they were enriched in some biological processes, such as the developmental process, cellular process, and metabolic process. These results further suggest that AS events play an important role during male chicken germ cell differentiation. An interesting finding is that the metabolic process was not only enriched in GO terms, but also highly enriched in KEGG pathways. Similarly, many reports conclude that metabolic changes accompany the SSC differentiation [50,51]. We suspect that AS events induce the pathway transmission during male germ cell formation via metabolic transform. In addition, the calcium signing pathway, a key player in cell differentiation and development, was found to be regulated by some spliced factors in our study [52,53]. Being aware of many pathways, such as Wnt, MAPK, and mTOR, involved in the chicken germ cell formation process, we observed that abundant genes within these crucial pathways exhibited AS events. The implication is that AS events may affect germ cell differentiation via these pathways [54,55,56]. Moreover, germ cell-specific genes such as NANOG, POU5F3, LIN28B, BMP4, STRA8, and LHX9, which act as stage markers of germ cells, were developmentally regulated and stage-specific. In our results, the expression of these genes was associated with AS events transmitted through male chicken germ cell differentiation [39,40,41,42,43,44]. AS events may regulate the expression of these genes and subsequently impact the differentiation and developmental cell process. Taken together, AS is a complex and important regulatory mechanism, which may play a crucial role during male germ cell differentiation.

## 5. Conclusions

In summary, we have provided a comprehensive atlas on the genome-wide scale of the AS event landscape in male chicken germ-line cells (ESCs, gPGCs, and SSCs) and presented the distribution of these AS events. AS events have reportedly been involved in the germ cell differentiation process since they were first discovered. This helps us understand how AS events are associated with the male germ cell differentiation process, and to possibly improve treatment options for male infertility in animals and humans.

## Figures and Tables

**Table 1:** Sequences of qRT-PCR primers.

Gene	Transcript ID	Primer (5′-3′)	Title 3
*NANOG*	ENSGALT00000044516	F: TACCCCAGACTCTGCCACTA	163 bp
		R: CTTCTGGCTCTGAAACCGC	
*POU5F3*	ENSGALT00000043197	F: GATGCGCCGACCTCAGAG	112 bp
		R: CATAGAGCGTGCCCAGAG	
*LIN28B*	ENSGALT00000044077	F: CAATGTGAGGATGGGCTTCG	220 bp
		R: ACTTCCTAAACAGGGGCTCC	
*BMP4*	ENSGALT00000020316	F: GATCTCTACCGGCTCCAGTC	174 bp
		R: GTTGAAGACGAAGCGGATCC	
*STRA8*	ENSGALT00000038151	F: TCCACGGCTATTTCACACCT	242 bp
		R: TCAAGGAAACCAGCAGCAAC	
*LHX9*	ENSGALT00000003480	F: GAACTCACCTGCTTTGCCAA	181 bp
		R: GAGTCTTGTTGCAGGTGGTG	
*GAPDH*	ENSGALT00000023323	F: TGGGAAGCTGTGGAGAGATG	166 bp
		R: GCAGGTCAGGTCAACAACAG	

**Table 2:** The identification of AS events in chicken ESCs, gPGCs, and SSCs.

AS Classification	ESCs		gPGCs		SSCs	
	AS Events	Gene	AS Events	Gene	AS Events	Gene
Alternative 5′ Splice Site (A5′)	16,741	14,560	18,697	12,639	19,391	13,402
Alternative 3′ Splice Site (A3′)	16,273	14,609	17,217	12,874	17,515	13,601
Exon Skip (ES)	2964	1412	7002	2487	7212	2605
Retained Intron (RI)	1476	649	3564	1271	3186	1169
Alternative Exon (AE)	1040	486	2475	995	2596	1113

## Data Availability

The Illumina reads are available in the Sequence Read Archive (SRA) database at NCBI under study accession number PRJNA608148. All data are presented in the main manuscript and are available to readers.

## Supplementary Materials

The following are available online at https://www.mdpi.com/article/10.3390/ani11051469/s1, Table S1: The AS event analysis in chicken ESCs, Table S2: The AS event analysis in chicken gPGCs, Table S3: The AS event analysis in chicken SSCs, Table S4: The total gene mRNA expression (FPKM) of ESCs, gPGCs, and SSCs, Table S5: The spliced gene mRNA expression (FPKM) of ESCs, gPGCs, and SSCs, Table S6: The GO term analysis of AS event genes during male chicken germ cell differentiation, Table S7: The KEGG pathway analysis of AS event genes during male chicken germ cell differentiation.

## Abbreviations

AS	Alternative Splicing
ESCs	Embryonic Stem Cells
gPGCs	Primordial Germ Cells
SSCs	Spermatogonia Stem Cells
AP	Alternative Promoter
AT	Alternative Terminator
ES	Exon Skipping
IR	Intron Retention
AE	Alternative Exon
FPKM	Fragments Per Kilobase Million
GO	Gene Ontology
KEGG	Kyoto Encyclopedia of Genes and Genomes
